# Influence of Chitin Source and Polymorphism on Powder Compression and Compaction: Application in Drug Delivery

**DOI:** 10.3390/molecules25225269

**Published:** 2020-11-12

**Authors:** Linda Al-Hmoud, Deeb Abu Fara, Iyad Rashid, Babur Z. Chowdhry, Adnan A. Badwan

**Affiliations:** 1 Chemical Engineering Department, School of Engineering, University of Jordan, Amman 11942, Jordan; l.alhmoud@ju.edu.jo; 2 Research and Innovation Centre, The Jordanian Pharmaceutical Manufacturing Company (JPM), P.O. Box 94, Naor 11710, Jordan; irashid@jpm.com.jo (I.R.); adnanbadwan@gmail.com (A.A.B.); 3 School of Science, Faculty of Engineering & Science, University of Greenwich, Medway Campus, Chatham Maritime, Kent ME4 4TB, UK; b.z.chowdhry@greenwich.ac.uk

**Keywords:** chitin, polymorphs, chitin sources, chitin characterization, disintegration, dissolution, crushing strength, compression analysis

## Abstract

The objective of the research reported herein is to compare the compaction properties of three different chitin extracts from the organisms most used in the seafood industry; namely crabs, shrimps and squids. The foregoing is examined in relation to their polymorphic forms as well as compression and compaction behavior. Chitin extracted from crabs and shrimps exhibits the α-polymorphic form whilst chitin extracted from squid pins displays a β-polymorphic form. These polymorphs were characterized using FTIR, X-ray powder diffraction and scanning electron microscopy. Pore diameter and volume differ between the two polymorphic powder forms. The β form is smaller in pore diameter and volume. Scanning electron microscopy of the two polymorphic forms shows clear variation in the arrangement of chitin layers such that the α form appears more condensed due to the anti-parallel arrangement of the polymer chains. True, bulk and tapped densities of these polymorphs and their mixtures indicated poor flowability. Nevertheless, compression and compaction properties obtained by applying Heckle and Kawakita analyses indicated that both polymorphs are able to be compacted with differences in the extent of compaction. Chitin compacts, regardless of their origin, showed a very high crushing strength with very fast dissolution which makes them suitable for use as fast mouth dissolving tablets. Moreover, when different chitin powders are granulated with two model drugs, i.e., metronidazole and spiramycin they yielded high crushing strength and their dissolution profiles were in accordance with compendial requirements. It is concluded that the source of chitin extraction is as important as the polymorphic form when compression and compaction of chitin powders is carried out.

## 1. Introduction

Chitin (CH; poly-β-(1,4)-*N*-acetyl glucosamine) occurs in the exoskeletons of crustaceans, insects and the cell walls of algae and fungi. CH has a protective and supporting function; it is responsible for the rigidity of the exoskeletons of insects and crustacean, and its presence in the cell walls of fungi and algae facilitates movement [1]. Currently, there are no synthetic methods for preparing CH. As a result, methods to obtain CH are dependent on extraction from crustaceans and algae. Usually, these methods are carried out in the following sequence: deproteinization, demineralization and discoloration [2]. The methods of preparation, as well as physical and chemical characterization of CH, are the subject of an extensive review by Daraghmah et al. [1].

Usually, CH exists in different organisms in its “complexed” forms with proteins, carbohydrates, or lipids. This means that CH, in order to interact with other chemical functional groups in the tissues of organisms, must have free functional binding groups. This is clear evidence that CH must have some deacetylated domains which means it cannot be isolated in pure form. Consequently, purification of CH involves freeing this insoluble material from various debris and other active matter such as deacetylated derivatives; namely, chitosan (CHS) and glucosamine. It seems that there is no clear demarcation between extraction and purification of CH which is therefore obtained with different molecular weights ranging from hundreds to thousands of kDa. Regardless of variations in molecular weight, CH can occur in three distinct existing polymorphic forms: α, β and γ. CH polymer chains in the α polymorph are aligned in an anti-parallel fashion, whilst in β polymorphs the alignment occurs in parallel. In the γ form the CH polymer chains are arranged in a pattern whereby two parallel chains are in one direction whilst the third is in the opposite direction [1]. Whether or not the anatomical variation of CH in a particular organism affects the mechanical properties of extracted CH is still the subject of active research.

CH is a polymer which is insoluble in water and other conventional solvents. It has a structure deprived of active functional groups. This leaves the CH surface without any reactive functional groups with no ability to react with any chemical moieties; thus, it is chemically inert. In addition, its lack of absorption in the human body allows it to be considered as a pharmacologically inert material. This means it is an ideal material to be used as a pharmaceutical excipient from both a chemical and pharmacological perspective [3,4]. Furthermore, CH is classified as grass in the FDA classification for materials safety usage [5]. This unique characteristic keeps the door open for future use as a pharmaceutical excipient particularly in liquid and solid dosage forms. The challenge in utilizing CH in solid pharmaceutical preparations is its powder flow and mechanical characteristics, including compaction and compression [6].

It may be advantageous to highlight the similarity of CH with the most popular excipient in pharmaceutical solid dosage form preparations, i.e., cellulose and its derivatives such as micro crystalline cellulose (MCC) [7]. MCC shares with CH its β-(1,4) glycosidic bond, and its relative chemical and pharmacological inertness. Researchers in the CH field are attempting to emulate and test if the same techniques which have been successfully used to modify MCC can be applied in the field of CH research, particularly in solid pharmaceutical dosage form preparations [8,9].

It is well known that, like MCC, CH and CHS powder flow properties suffer from low bulk density. This causes unsmooth flow which is attributed to the fibrous nature of these natural polymers [6,10]. Attempts to alleviate this drawback have been tested by adding silicon dioxide to CH and CHS powders [11,12]. The addition of silicon dioxide facilitates smooth flow in tableting machines. Furthermore, combining CH/CHS with Avicel PH201, starch 1500, calcium carbonate or gelatin have been tested and were found to dramatically improve the flow behavior of the composite powders [13,14,15]. The improvement in flow properties has encouraged novel applications of these composites in producing pharmaceutical solid dosage forms excipients, especially as direct compression excipients. It is worth mentioning that various attempts to utilize CH as a novel solid drug delivery system were carried out by Daraghmeh et.al. [16], whereby CH was co-processed with mannitol to produce orodispersible tablets able to disintegrate in the mouth within a few seconds. Furthermore, CH was formulated, by Gana et.al. [17], with cephalosporins and metal silicates in order to obtain an insight into the effect of pH on the CH surface and its influence on drug stability. Abu Fara et al. [8] used roller compaction to improve CH powder flow qualities and to make its compression and compaction characteristics compatible for use in industrial pharmaceutical machines. Additionally, there are some on-going trials to extend the use of CH and CHS as excipients in a similar method followed in improving MCC powder flow using spray drying [6,18]. Such studies, show that CH is a suitable solid pharmaceutical excipient. As a result, it can be concluded that CH powder modification is essential in order to commercialize this novel excipient.

Natural polymers usually contain crystalline and amorphous domains in their structure. The balance between these two components controls the powder flow behavior. It has been reported that during the compaction of MCC, its crystalline domains are responsible for fragmentation while its amorphous domains are responsible for polymer plasticity, which induces bonding [7]. This is confirmed in the case of lactose, whereby amorphous lactose offers better compactibility than the crystalline form [19,20]. Accordingly, it seems that an amorphous powder is more compactable but the bulk density is usually lower than the crystalline material. Consequently, studying the impact of the crystalline-amorphous balance on powder flow is a pre-requisite to its industrial use. However, a review of the scientific literature does not show if CH polymorphs differ in their response towards compression and compaction.

CH has diverse functions in different tissues in an organism; e.g., the CH in the lining of the gastrointestinal tract in some organisms is more flexible to suite its functional role and has more elasticity than exoskeleton CH, which is a harder material. Such functionality raises the question whether CH polymorphs behave in a similar manner when compressed, or does the functional use dictate their mechanical strength, as expressed in different tissues of organism? As sea food remains from industrial packaging contain a mixture of CH with various origins, it would be interesting to compare extracted CH from the most utilized CH sources, namely shrimp, crab and squid. These organisms are known to have α (shrimp and crab) and β (squid) CH polymorphs. Such polymorphs can be differentiated by their powder X-ray diffraction patterns. It would be interesting to test each polymorph individually and discern the differences in their compaction and compression behavior.

The present work focuses on pharmaceutical powder compression and compaction properties of CH extracted from the most widely used sea food organisms namely crabs, shrimps, and squids. The influence of variation in α and β CH polymorphic forms due to CH extraction source on compression and compaction is explored. Hence it may be possible to ascertain whether CH sources must be processed separately or collectively prior to their extraction.

## 2. Results

### 2.1. Bulk, Tapped Density and True Density

The bulk, tapped, and true densities of CH from different sources, as well as of their mixtures (Sh9Sq1, Sh7Sq3, and Sh5Sq5) are presented in Table 1. Crab (CH-Cr) and shrimp (CH-Sh) CHs have much higher bulk and tapped densities than squid CH (CH-Sq).

To study the effect of mixing CH from different sources, CH-Sh was chosen as representative of α-CH, and mixed with CH-Sq (β-CH) in different compositions, as shown in Table 1. With respect to bulk and tapped densities, such mixtures manifested intermediate values between the low (CH-Sq) and high (CH-Sh) bulk and tapped densities.

In comparing the powder flowability of CH from different sources, the Carr index (CI) and Hausner ratio (HR) were determined [21]. Flowability results, as illustrated in Table 1, indicate that CH from different sources, as well as their mixtures, exhibit extremely poor flowability. Flow interpretation criteria based on HR and CI is given in the literature [21]. As for densities, the mixtures manifested intermediate HR and CI values between the low and high values presented by CH-Sh and CH-Sq, respectively.

### 2.2. Water Content

The water content of CH from different sources are presented in Table 2. The water content of CH-Cr and CH-Sh is almost half that of CH-Sq.

### 2.3. Porosity of Powders

The porosity parameters of the three CH powder samples are shown in Table 3. The pore volume of crab and shrimp CHs are of similar values, whereas that of squid CH is ~20% less. On the other hand, crab CH has the largest pore diameter and squid CH has the smallest.

### 2.4. FTIR

**Figure 1:**
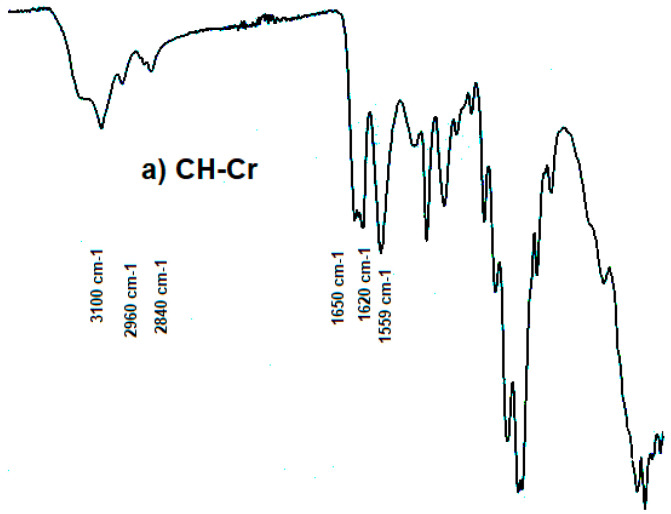

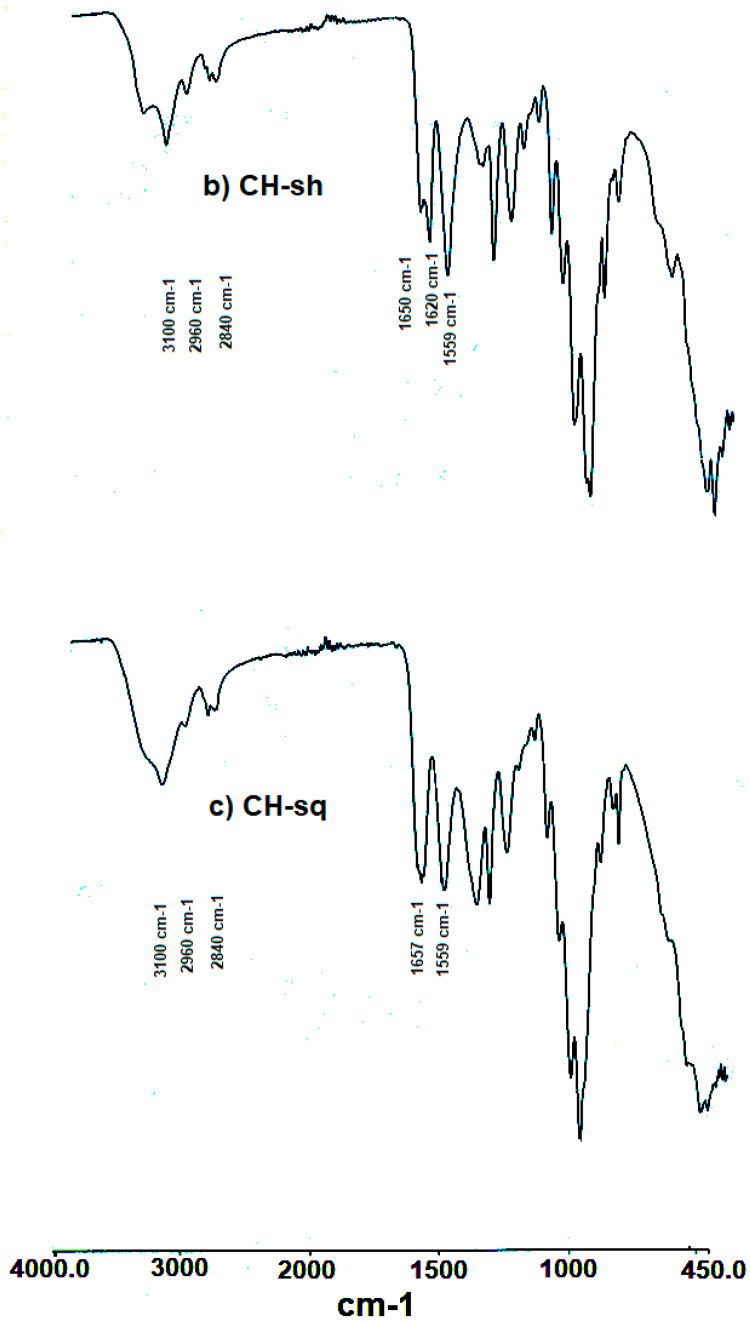
FTIR spectra of (**a**) CH-Cr, (**b**) CH-Sh (**b**), and (**c**) CH-Sq.

The Fourier-transform infrared (FT-IR) spectra of α-CH (CH-Cr and CH-Sh) and β-CH (CH-Sq) are shown in Figure 1. For α-CH, the amide I band is split at about 1650 and 1620 cm^−1^ (Figure 1a,b), whereas it is a single sharp band at about 1657 cm^−1^ for β-CH (Figure 1c). The amide II band appears at about 1555 and 1559 cm^−1^ for α- and β-CH, respectively. Both polymorphs show strong absorption bands in the 3100–3285 cm^−1^ region which correspond to the N–H group. Bands in the 2840–2960 cm^−1^ region are due to CH, CH_2_, and CH_3_ in both CH polymorphs [1].

### 2.5. X-ray Powder Diffraction (XRPD) Analysis

**Figure 2:**
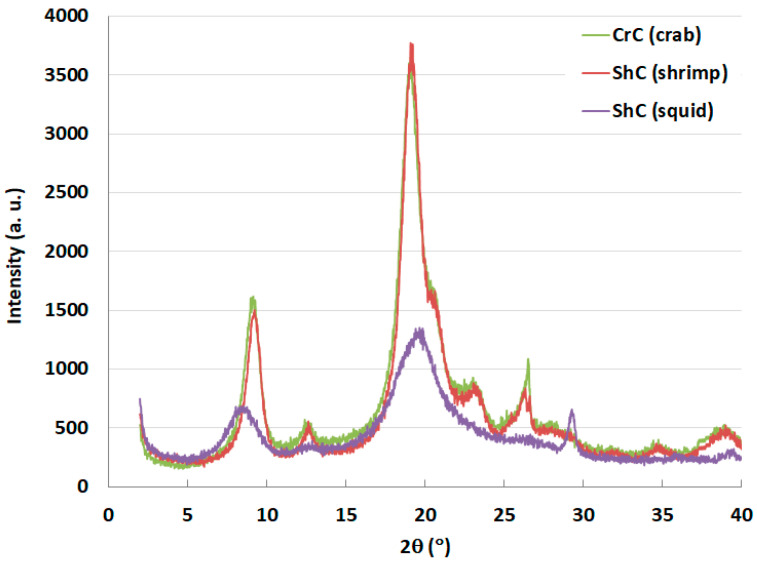
X-ray powder diffraction (XRPD) patterns of CH-Cr, CH-Sh, and CH-Sq CH.

The XRPD profiles of CH from different sources are presented in Figure 2. The XRPD patterns of crab (CH-Cr) and shrimp CH (CH-Sh) show four sharp crystalline reflections indicative of α-CH at 2θ = 9.3, 19.1, 20.6, and 23.2°.

The XRPD pattern of squid CH (CH-Sq) shows two broad crystalline peaks at 8.7 and 19.8° which indicates that squid CH is of the β-CH type. The crystal size of CH-Cr and CH-Sh is almost the same. Squid CH has a smaller crystal size than crab and shrimp as its pattern peaks at 9° and 19° are much wider for CH-Sq than the corresponding ones for CH-Cr and CH-Sh. Table 4 shows the crystalline index (*I*_CR_) of the three CH samples. These results indicate that α-CH has a more crystalline structure because of its inter-sheet and intra-sheet structure [22,23]. Crystalline index (*I*_CR_) is used to estimate the degree of deacetylation (DDA) of CH using a method reported by Zhang et al. [24]. DDA results presented in Table 4 shows that α-CHs (CH-Cr and CH-Sh) is less deacetylated than β-CH (CH-Sq), which conforms with findings reported in the literature [1,2,22].

### 2.6. Scanning Electron Microscope (SEM)

**Figure 3:**
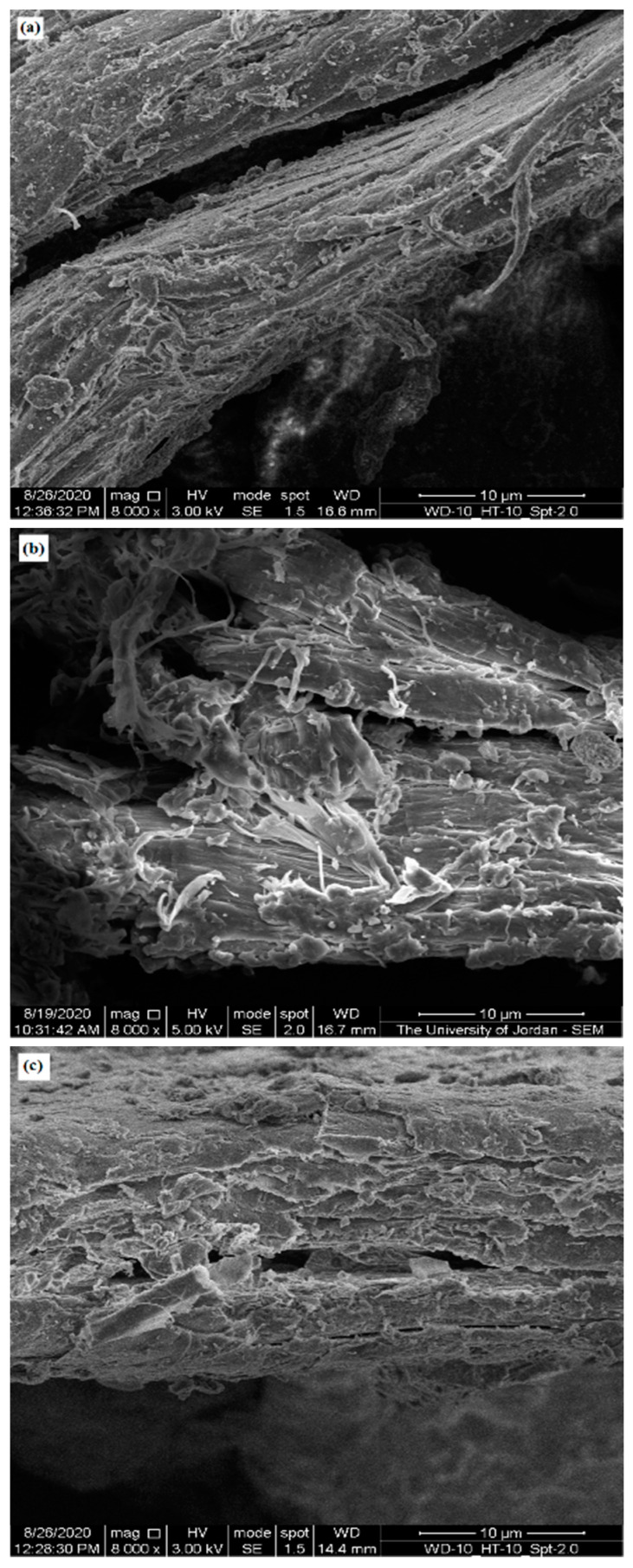
SEM image of (**a**) crab (CH-Cr), (**b**) shrimp (CH-Sh), and (**c**) squid (CH-Sq) CHs at 8000× magnification.

**Figure 4:**
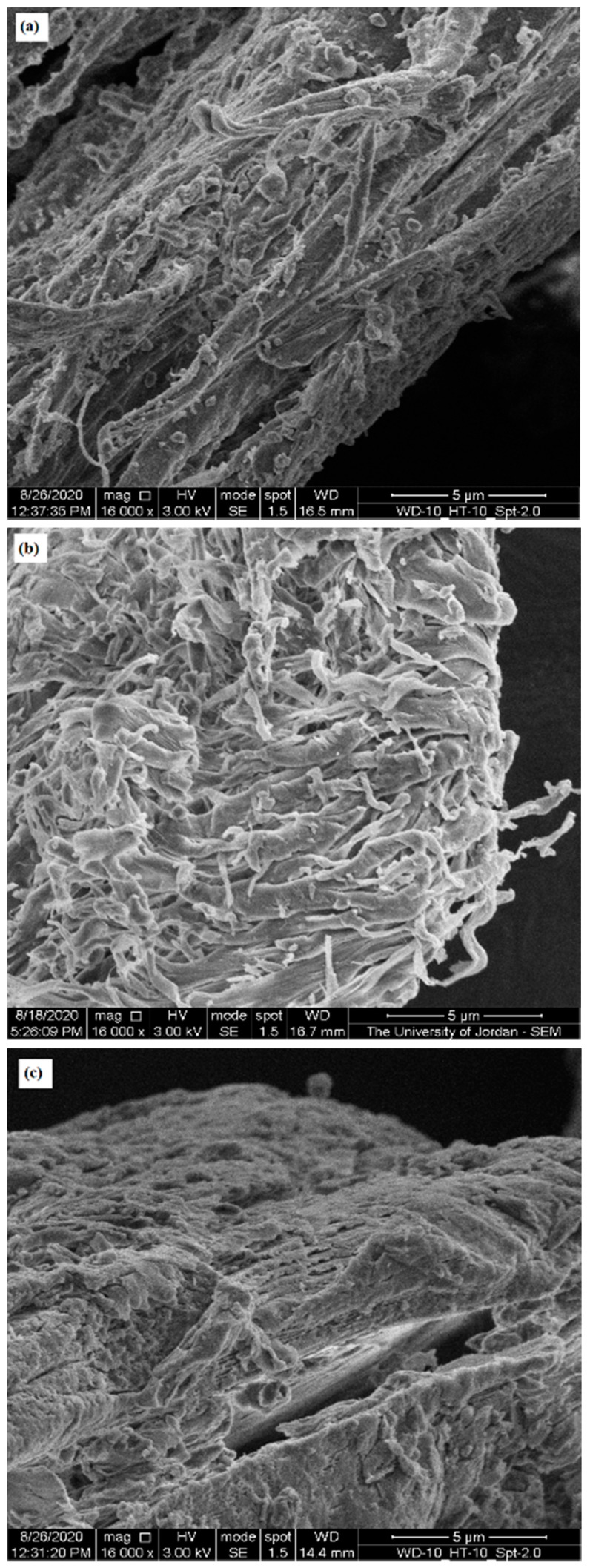
SEM image of (**a**) crab (CH-Cr), (**b**) shrimp (CH-Sh), and (**c**) squid (CH-Sq) CHs at 16,000× magnification.

**Figure 5:**
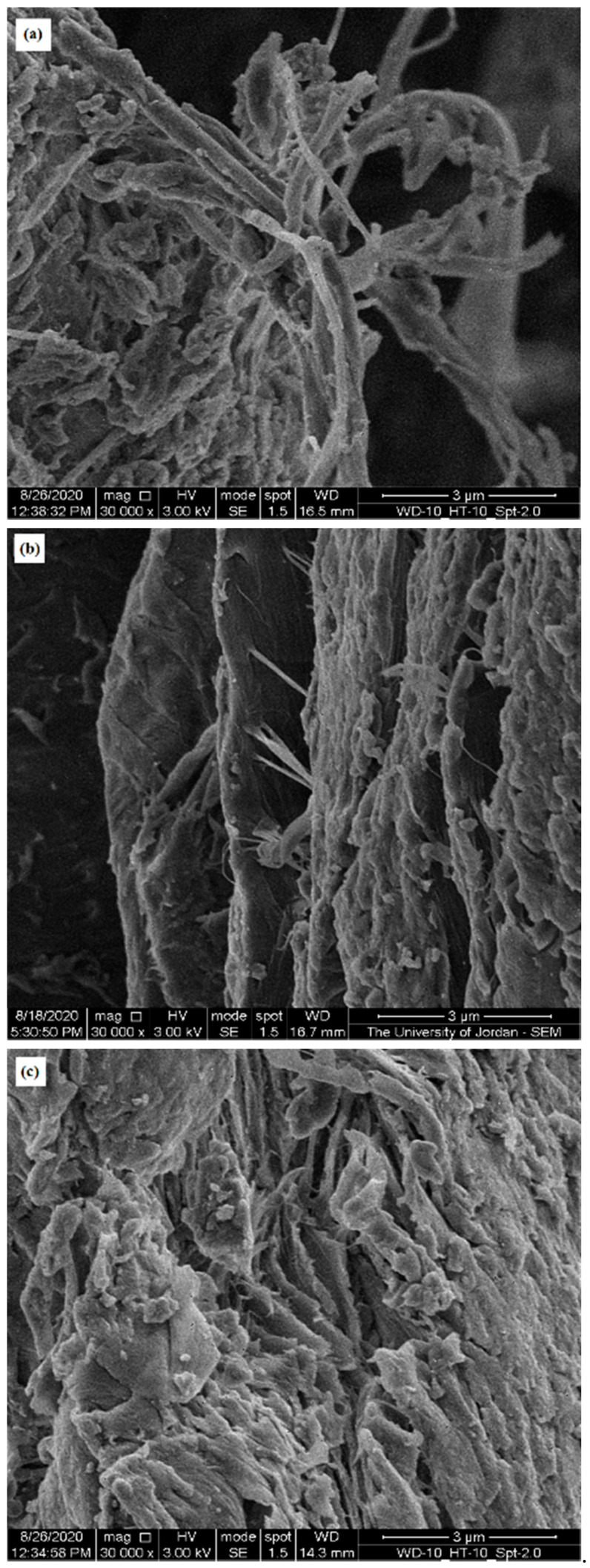
SEM image of (**a**) crab (CH-Cr), (**b**) shrimp (CH-Sh), and (**c**) squid (CH-Sq) CHs at 30,000× magnification.

**Figure 6:**
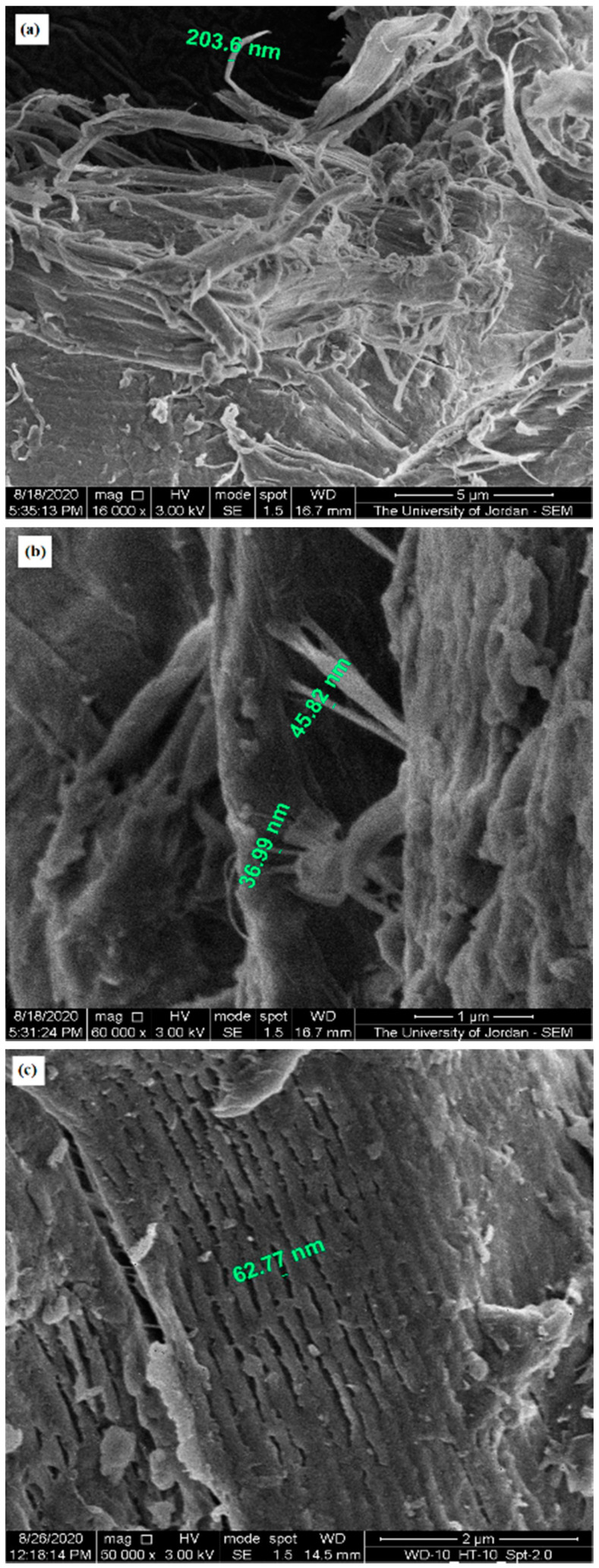
SEM image with measurement of fiber thickness of (**a**) shrimp (CH-Sh) CH at 16,000× magnification, (**b**) shrimp (CH-Sh) CH at 60,000× magnification, and (**c**) squid (CH-Sq) CH at 50,000× magnification.

SEM was used to highlight the contribution of micro-irregularities to powder physical properties by visualizing the surface of CH particles from different sources. Figure 3, Figure 4 and Figure 5 present SEM images of crab (CH-Cr), shrimp (CH-Sh), and squid (CH-Sq) CHs at a magnification of 8000×, 16,000×, and 30,000×, respectively. Figure 6 presents SEM images with measurement of fibers thicknesses of shrimp (CH-Sh) CH at 16,000× and 60,000× magnifications, and of squid (CH-Sq) CH at 5000× magnification.

At 8000× magnification (Figure 3) it is difficult to see any significant difference amongst the different CH samples. At 16,000× magnification (Figure 4) it can be noticed that squid chitin fibers are more organized than crab and shrimp CH samples. At 30,000× magnification (Figure 5) shrimp CH showed two types of fibers; one forming the layers, and the other connecting the layers. Squid CH layers have no connecting fibers.

The data in Figure 6 shows that the fibers forming shrimp CH layers have a thickness of ~200 nm (Figure 6a), where the layers are about 1 μm apart, and the fibers connecting them have a thickness of 35–45 nm (Figure 6b). On the other hand, squid chitin layers are around 60 nm apart and no connecting fibers can be observed at ×50,000 magnification (Figure 6c).

### 2.7. Compression Analysis

**Figure 7:**
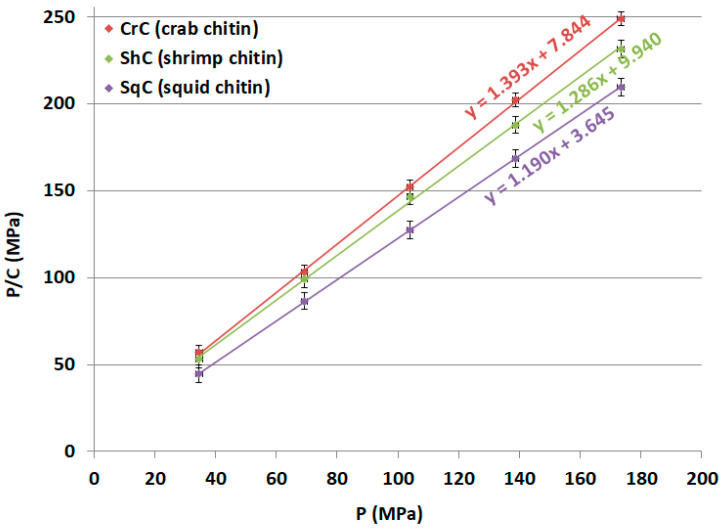
Kawakita plots of crab (CH-Cr), shrimp (CH-Sh), and squid (CH-Sq) CHs.

**Figure 8:**
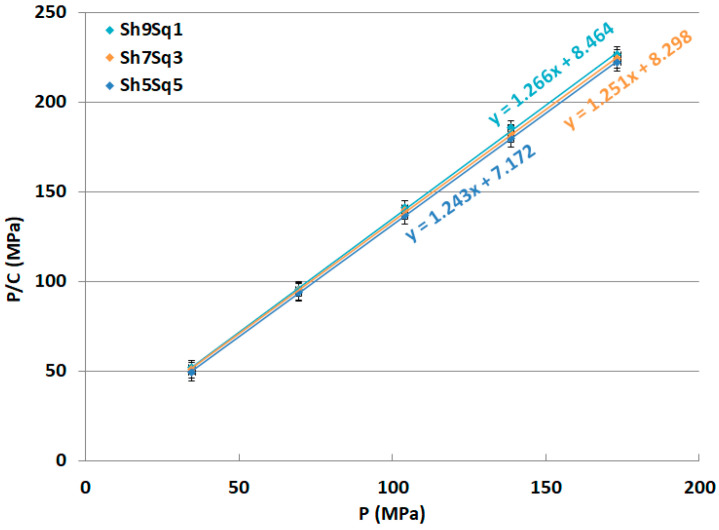
Kawakita plots of CH-Sh9Sq1, CH-Sh7Sq3, and CH-Sh5Sq5 CH mixtures.

The three main parameters (*a*, *P_k_* and *ab*) obtained via Kawakita analysis (as illustrated in Section 4.2.7. Compression Analysis) were analyzed in an attempt to interpret the compression behavior of the three samples of CH and their mixtures. The data in Figure 7 shows the Kawakita plots of CH-Cr, CH-Sh, and CH-Sq CHs, and Figure 8 shows the Kawakita plots of shrimp/squid CH mixtures; CH-Sh9Sq1, CH-Sh7Sq3, and CH-Sh5Sq5. Table 5 presents the values of Kawakita parameters for the different chitin types and their mixtures.

The maximum volume reduction that can be attained (*a*) illustrates that CH-Cr has the lowest *a* value with CH-Sq having the highest volume reduction when a compression force is applied. In addition, the results of the three mixtures show that the volume reduction value increases with increasing percentage of squid CH in the mixture.

*P_K_*, which represents the pressure needed to reduce the value of (*a*) to half its initial value, is the most important Kawakita parameter to be tested. This is because it represents how hard the CH granules are, and therefore their ability to be used in direct compression applications. Table 5 shows that the *P_K_* value of shrimp CH (CH-Sh) is the highest among the three types of CH. *P_K_* value of mixture containing shrimp and squid CHs at different fractional content; CH-Sh9Sq1, CH-Sh7Sq3, and CH-Sh5Sq5, decreases with increasing squid CH fraction.

The last Kawakita parameter used in this work to describe the compression behavior is *ab*. This parameter gives an indication of the degree of rearrangement of powder particles. As Table 6 shows, squid CH (CH-Sq) has the highest the extent of particle rearrangement (*ab*) upon compression. The value of *ab* is the lowest for shrimp CH (CH-Sh). The data in Table 6 also indicates that mixtures containing shrimp and squid CHs in different fractions; CH-Sh9Sq1, CH-Sh7Sq3, and CH-Sh5Sq5, show *ab* values that increase with increasing squid fraction of CH.

**Figure 9:**
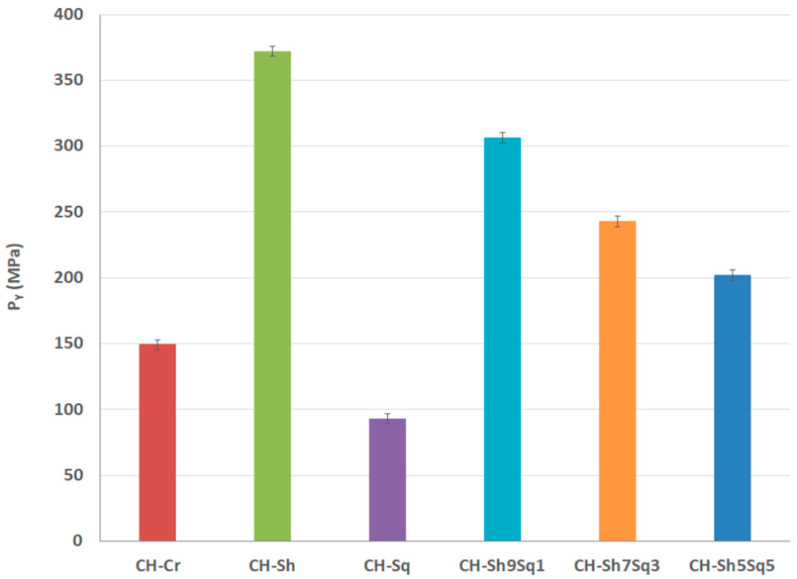
Heckel parameter (*P_Y_*) for CH-Cr, CH-Sh, and CH-Sq and their mixtures.

Compression analysis was further examined using the empirical Heckel model of compression analysis. In this model, the yield pressure (*P_Y_*) represents a critical outcome whereby it reflects the type and extent of deformation, i.e., plastic/elastic or brittle-fracture. The data in Table 6 and Figure 9 illustrates the yield pressure values for all tested powders. Results show that *P_Y_* ranking of pure CHs follows the order: shrimp chitin > crab chitin > squid CH. Adding squid CH to shrimp CH reduced the *P_Y_* value, and the more squid CH content in the mixture, the lower its *P_Y_* value: CH-Sh9Sq1 > CH-Sh7Sq3 > CH-Sh5Sq5.

### 2.8. Work of Compression

**Figure 10:**
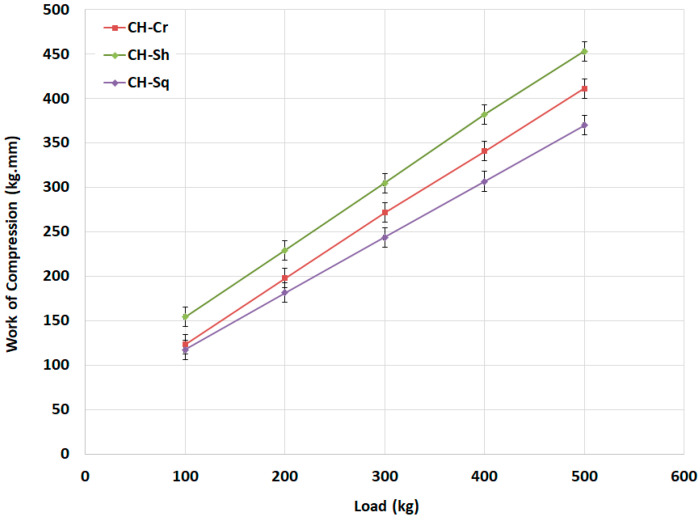
Compression work for CH-Cr, CH-Sh, and CH-Sq CHs.

**Figure 11:**
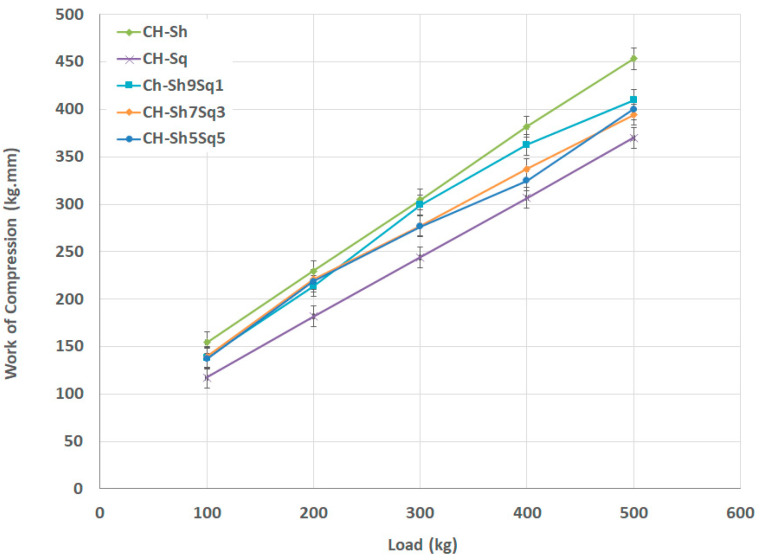
Compression work for CH-Sh9Sq1, CH-Sh7Sq3, and CH-Sh5Sq5 CH mixtures in comparison with pure CH-Sh and CH-Sq.

When powders of different types are compressed using the GTP, the instrument displays the force displacement curve during the descending/compression and decompression of the powders. The work of compression (*W_C_*), which is the area under the compression curves of the of the F-D profiles, is shown in Figure 10 and Figure 11. *W_C_* values were calculated at each compression force used. The results shown in Figure 10 indicate that at all loads, the *W_C_* of shrimp CH (CH-Sh) > *W_C_* of crab CH (CH-Cr) > *W_C_* of squid CH (CH-Sq).

The data in Figure 11 demonstrates the effect of adding β-CH (CH-Sq) to α-CH (CH-Sh) in different fractional contents. Mixtures containing different amounts of β-CH have *W_C_* values close to each other at low compression loads (100 and 200 kg), while at higher loads *W_C_* decreases with increasing the amount of added CH-Sq.

### 2.9. Tablet Crushing Strength

Tablet crushing strength for CH from different sources are presented in Table 7. CH-Cr and CH-Sh tablet crushing strength is almost half that of CH-Sq tablet crushing strength. The crushing strength of the mixture is closer in value to that of CH-Sq.

### 2.10. Tablet Disintegration

All tablets have recorded disintegration times of less than 10 s irrespective of CS source or mixtures comprised thereof.

### 2.11. Characterization of Metronidazole/Spiramycin Tablets Comprising Drug/CH

**Figure 12:**
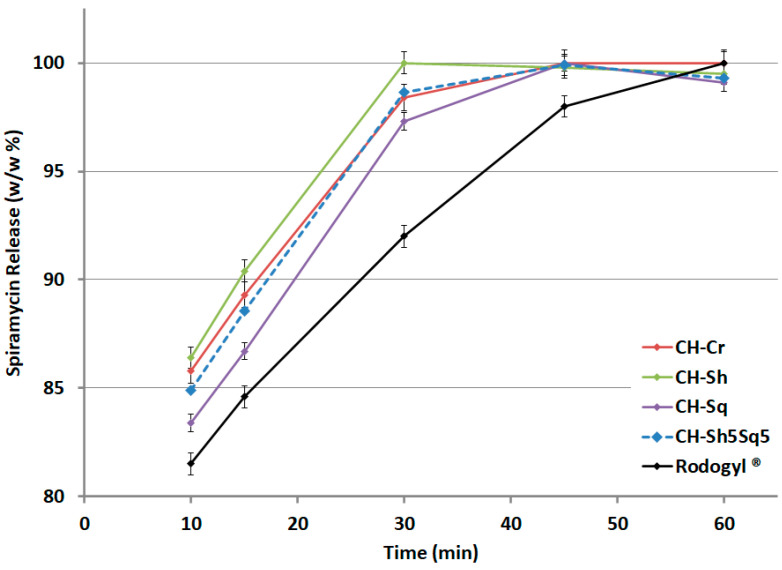
Dissolution profile for metronidazole (200 mg) tablets comprising compacted CHs or CH mixture (CH-Sh5Sq5) compared to Rodogyl^®^ tablets.

**Figure 13:**
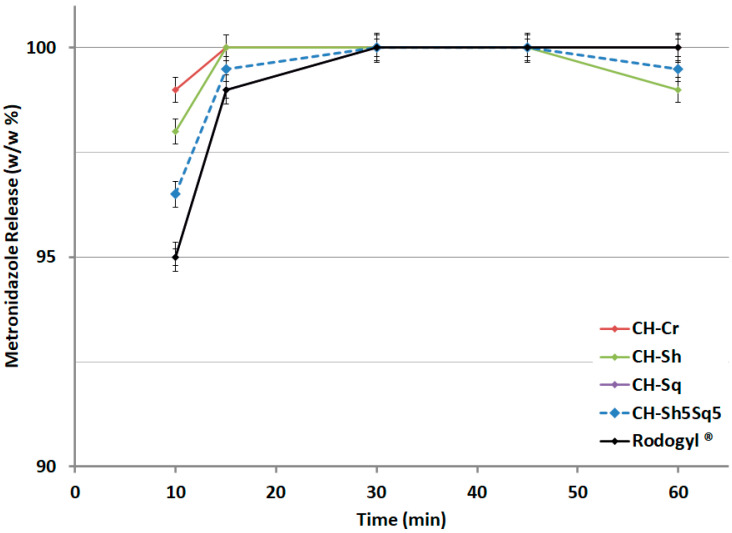
Dissolution profile of spiramycin (0.75 M UI) tablets comprising compacted CHs or CH mixture (CH-Sh5Sq5) compared to Rodogyl^®^ tablets.

Tablet dissolution data are illustrated in Table 8 and in Figure 12 and Figure 13. Figure 12 demonstrates that full drug release of metronidazole/α-CH (crab and shrimp) matrix is achieved within 15 min of dissolution time. This is faster than tablets comprising metronidazole/β-CH (squid) matrix which achieved full drug release within 30 min of dissolution time. Tablets comprising metronidazole/CH mixture (50 wt% shrimp and 50 wt% squid) as well as Rodogyl^®^ tablets also achieved full drug release within 30 min of dissolution time.

Results presented in Figure 13 demonstrates that full drug release of spiramycin/shrimp CH matrix was achieved within 30 min of dissolution time, and within 45 min of dissolution time for the other types of CH, as well as for CH mixtures. This was faster than Rodogyl^®^ tablets which achieved full drug release within 60 min of dissolution time.

## 3. Discussion

The prime objective of the present investigation was to test CHs extracted from different sources to assess their similarity and suitability for use as a future excipient in pharmaceutical solid dosage form preparations. The final goal is to reach a conclusion whether remains from the sea food packaging industry need to be classified according to their source prior to CH extraction process. This concern becomes evident when one screens the different scientific literature on CH, where there are indications that CH from different sources are chemically similar, but their mechanical properties vary [25].

The strategy followed in this work was to compare CH from crabs and shrimps, both of which belong to α polymorph, in order to find out if the variation in CH source has any influence on the extracted polymer compression and compaction properties. Furthermore, a comparison between α and β-CH polymorphs extracted from squid pins, regarding their compression and compaction behavior, was also conducted. Mixtures of the two polymorphs were also prepared, and their physical and mechanical properties were studied. Whether or not these polymorphic forms could facilitate compaction of two model drugs, namely metronidazole and spiramycin, which are difficult to compress on their own, was tested.

It is well known that shrimps, crabs and squids are the most used organisms in the sea food packaging industry [26]. These organisms differ in their species and their extracted CH is confined to the three well characterized and known polymorphs; α, β and γ. It is essential to first confirm this primary information. The Fourier-transform infrared (FT-IR) spectra of CH-Cr, CH-Sh, and CH-Sq are shown in Figure 1. Distinct vibrational bands appear at a wavelength of 1620–1660 with a distinct split in the band for α-chitin (Figure 1a,b), while there is a single sharp band for β-CH (Figure 1c). This confirms different previous reports on CH of α and β polymorphs [23,27]. Further confirmation of the polymorphic type was carried out using X-ray powder diffraction, Figure 2. The γ polymorph is scarce due to its limited occurrence in the lining of the digestive system of different organisms such as squid. Consequently, the present investigation was limited to a comparison of the compression and compaction behavior of the most commercially available two polymorphs, α and β. Such a comparison helps to clarify if the source of CH extraction causes variation in their related mechanical properties when compacted.

In addition to identifying polymorphic forms of molecules, XRPD has been used as a tool to shed light on the crystallinity and arrangement of CH in different polymer fibers. Figure 2 confirms α polymorph in CH-Cr and CH-Sh, and β polymorph in CH-Sq. The crystalline index, Table 5, shows that the α polymorph is more crystalline than the β polymorph; 84–85% in CH-Cr and CH-Sh, and 73% in CH-Sq. SEM images, Figure 3, Figure 4, Figure 5 and Figure 6 show the parallel arrangements of β-CH sheets with clear spacing. This is indicative of their ability to hydrate and gel much more than α polymorph [23,27]. Interestingly, the β polymorph shows a smaller pore size and pore volume which may indicate that the value of the parameter a (the capillary water effect) may contribute to its functional strength and flexibility in squid pins. Indeed, the water content determination of CH-Sq shows almost double the value of Ch-Cr and CH-Sh (Table 3). This dramatic variation in water content is an important difference which can be beneficial in the use of CH as excipients for pharmaceutical solid dosage forms. Water content results are consistent with the fact that β-CH (CH-Sq) is more susceptible to intra-crystalline hydration than α-CH (CH-Cr and CH-Sh) due to its lack of intra-sheet hydrogen bonds [23].

Bulk densities of the three types of CH (Table 2) are different although shrimp and crab are similar in polymorphic form. The difference between shrimp and crab CH measured bulk densities may be due to a difference in material arrangements in nature in those living organisms depending on their functional use. Such variation has been previously reported, where extracted CHs from male and female grasshoppers show some differences in CH content and surface morphology [27]. Accordingly, one would expect to observe some variations in CH extracted even from the same species. The higher crab and shrimp chitin bulk densities indicate that these powder samples comprise denser aggregated particles. Such aggregation could be a result of particle-particle adherence which may be attributed to the highly fibrous structure of CH-Cr and CH-Sh, whereas CH-Sq displays a structure which is much less fibrous (Figure 5 and Figure 6). The plain surface of CH-Sq with no fibrous extensions is responsible for the absence of an interlocking structural network, which consequently leads to the formation of aggregated particles in CH-Cr and CH-Sh.

As expected, all types of CH did not provide good flowability as indicated by their HR and CI values (Table 2), which is attributed to their low bulk densities. The foregoing has led, various workers, to suggestion that these powders be treated by roller compaction or slugging before using them as excipients [8]. Thus CH-Sh would be more favorable for tablet compression than CH-Cr and CH-Sq since it has the lowest HR and CI values.

Parameters from Heckle and Kawakita equations indicate that shrimp powder is more resistance to compression than the other two sources. This is reflected in the force required for compression (*P_K_*) which is significantly higher in value than for the other two CH samples (Table 6). Such variation makes the work required for compression higher, as emphasized in the data in Figure 10. Indeed, this is a real indication that differences in CH properties, such as bulk and tapped densities, are reflected in the compression forces required to convert the powders into compacts. The mechanical properties of CH are similar to cellulose derivatives e.g., micro crystalline cellulose (MCC), where reports have shown that various cellulose polymorphs behave differently in response to compaction forces. MCC requires a disintegration agent when used in tablet manufacturing whilst CH powders are self-disintegrating. This is advantageous in terms of the use of CH for pharmaceutical dosage forms which need to be dissolved in the buccal cavity.

The three parameters extracted from the Kawakita equation, namely *a*, *ab*, and *P_K_*, are interesting. CH from different sources, which show differences in particle morphology and crystallography, has concurrently shown differences in powder compression behavior. Among the three types, CH-Sq (β-CH) displays the highest volume reduction (*a*), particle rearrangement (*ab*), and the lowest compression force (*P_K_*) to reduce the powder bed volume (Table 6). In other words, in order to produce a compact, CH-Sq requires the smallest force to yield the highest volume reduction in comparison with CH-Cr and CH-Sh (α-CHs). This may be attributed to the anti-parallel and parallel arrangement of α- and β-polymorphs, respectively. As a matter of fact, CH-Cr and CH-Sh responses to compression vary although they belong to the same polymorph. This may be due to variation in the mechanical strength of the extracted materials. Concurrently, examining the results from Heckel analysis (Table 7) CH-Sq is the highest plastically deforming material, it can be stated that compression of the less fibrous and less aggregated material, i.e., CH-Sq, is more mechanically engaged in the deformation of the highly plastic material than the more fibrous one, i.e., CH-Cr and CH-Sh. Such a high extent of deformation shown by CH-Sq contributes to the high reduction of its powder bed volume upon compression compared to CH-Cr and CH-Sh. Moreover, the high extent of deformation of CH-Sq enables the appearance of fresh new surfaces for surface-to-surface contact and bridging [28,29]. Thus, compacts made of CH-Sq have a higher crushing strength than compacts made of CH-Cr and CH-Sh (Table 8).

Although the α-polymorph is more resistant to reduction in size compared to the β-polymorph, such behavior does not hinder both polymorphs from possessing an excellent crushing strength and very short disintegration time. This means direct compression of these individual powders can be utilized in preparing tablets or films which can dissolve in the mouth within seconds liberating the active content. This property can be highly advantageous and utilized in oral dissolving tablets, which are required to dissolve fast but at the same time be composed of a hard compact. The extracted CH polymorphs fit very well for such a function and future research potentially bodes well in this direction. However, due to its fast dissolving characteristics, CH can be used as a disintegrating agent; indeed, this has been previously reported [1,11]. Both polymorphs can be used as direct compression excipients in pharmaceutical formulations, either as whole or partially, and their need for a driving force for compaction is low, allowing ease of use in tableting machines.

Bearing in mind that the work/energy of compression is the product of force and displacement, CH-Sq manifested the lowest energy needed for compression (Figure 10), since it manifested the lowest compression force (or lowest *P_K_*) needed to produce the hardest compacts (Table 6 and Table 8). Irrespective of the fact that hard compacts can be made using CH-Sq, a disintegration time of less than one minute, like other CH sources, makes the super-disintegration power of CH independent of tablet hardness.

The dissolution performance of formulations containing CH from different sources was studied and compared with Rodogyl^®^ formulation, which contains a superdisintegrant and a filler with disintegrant action [29] in addition to two drugs: spiramycin and metronidazole. The drug release profile for the two drugs formulated with CH-Cr, CH-Sh, and CH-Sq and the mixture CH-Sh5Sq5 showed that 8.3% CH was sufficient to attain faster and complete drug release compared to Rodogyl^®^, for both spiramycin and metronidazole (Figure 12 and Figure 13). However, the release of the two drugs in formulations containing CH-Sq was slightly slower than that in formulations containing CH-Cr and CH-Sh. Nevertheless, the mixture containing CH-Sh5Sq5 speeded up the drug release from CH-Sq formulations for both drugs. This makes it necessary to use more than one source of CH in the drug formulations. Thus, a combination of CH sources is recommended to attain optimized tablet physical properties and optimum drug release profile.

Mixtures of CH polymorphs demonstrate an optimized action when compacted. This results in a satisfactory mixture suitable to be compressed and compacted. At first glance, this may suggest to gather remains from sea food packaging industry without classification, but such process shall yield CH with different characteristics depending on the percentage of squid remains involved in extraction process. Such operational procedure will result in lack of consistency in the extracted CH in each produced batch, which would in turn influence the manufactured dosage forms. Consequently, separating the organisms before CH extraction would yield a homogeneous CH powder, enabling pharmaceutical formulator to take advantage when formulating dosage forms by adding the required polymorph in the formulation stage depending on required properties. Alpha polymorph CH is reasonably less expensive than beta polymorph which reserve this polymorph for certain required formulation functions. Indeed, this polymorph can be used to form tablets or films with excellent hardness capable of withstanding mechanical vibrations encountered throughout manufacturing, packaging and transferring steps. The main advantage of CH as an excipient is its ability to form hard compacts with a very short disintegration time. This gives this excipient an advantage compared to excipients based on cellulose derivatives, without any need to add a disintegrating agent to formulations containing CH. Thus, CH is a suitable future excipient. Differences in sources or polymorphic form extend its usage particularly as a solid dosage form excipient suitable for drugs required to be released in a very-short time. CH must be extracted using a single species process, whereby the extracted CH has a well-defined function.

As a general remark, these raw excipients must be exposed to a process of compaction; for example, by using roller compaction, as has been previously reported by our group [8]. The variation in compaction properties between different sources of CH and their mixtures suggest that separation of sources yielding different polymorphs has an advantage in producing excipients with functional properties e.g., as a disintegrant or as filler. CH is chemically and pharmacologically inert and can be considered as a future pharmaceutical excipient.

## 4. Materials and Methods

### 4.1. Materials

CH powder from shrimp (CH-Sh) was obtained from G.T.C. Bio Corporation (Qingdao, China). The powder was sieved and the portion of particle size less than 90 μm was used for further analysis, characterization and testing. CH samples from crab (CH-Cr) and squid (CH-Sq) were also obtained from G.T.C. Bio Corporation in the form of flakes and fibers, respectively. They were ground using a ring mill, the powder was sieved and the portion of particle size less than 90 μm was used for further analysis, characterization, and testing. To study the effect of mixing chitin from different sources, CH-Sh was chosen as representative of α-chitin, and mixed with CH-Sq (β-chitin) using the compositions shown in Table 9; these mixtures were characterized and analyzed.

### 4.2. Methods

#### 4.2.1. True, Bulk, and Tapped Density Measurement, and Flow Determination

The bulk density of chitin powder samples (shrimp, crab and squid) in g/mL was measured by pouring the powder into a 25 mL volumetric cylinder. The bulk density of all samples was calculated as the ratio of the mass over the volume it occupies. Tapped density measurements were carried out by physical tapping of the cylinder for 100 mechanical taps then dividing the mass over the tapped volume. The cylinder was tapped again for 200 mechanical taps. If the decrease in volume (*V*_100_–*V*_200_) was less than 2 mL then the *V*_200_ was considered. If the difference was greater than 2 mL, the increments are repeated, such as the 200 taps, until the difference between succeeding measurements was less than or equal to 2 mL.

The reduction in powder bulk volume due to tapping is considered to be an indication of powder flowability which was evaluated by the Hausner ratio (HR) and Carr Index (CI). As HR and CI increase in value, the flowability is reduced.

HR is calculated using Equation (1), and CI is calculated using Equation (2):

(1) $$\mathrm{HR}=\frac{\rho_{tapped}}{\rho_{bulk}}$$

(2) $$\mathrm{CI}=100\times \left(\;\frac{\rho_{tapped}\;-\;\rho_{bulk}}{\rho_{bulk}}\right)$$

Flow interpretation criteria based on HR and CI is given in the literature [21]. True density was determined by fitting compaction data, i.e., compaction pressure versus tablet density, according to the method reported by Sun [30].

#### 4.2.2. Water Content Determination

CH samples were analyzed for water content by placing the samples in a porcelain crucible and drying them in a conventional oven at 105 °C to constant weight.

#### 4.2.3. Determination of the Porosity of Powders

Brunauer-Emmett-Teller (BET) pore volume, and pore diameter were determined by physical adsorption of nitrogen gas using a Nova 2200 multi-speed high gas sorption analyzer (version 6.11, Quantachrome Co., Syosset, NY, USA). Samples were subjected to nitrogen gas for adsorption under isothermal conditions at 77 K. The samples were initially placed in a vacuum oven at 60 °C for 24 h. An empty reference cell (Sartorius, analytic, A120s, Göttingen, Germany) and the sample (~500 mg) were placed in the chambers.

#### 4.2.4. FTIR Spectrophotometry

IR spectrophotometry was carried out using Perkin Elmer Spectrum Two UATR FTIR spectrometer (Akron, OH, USA) with a resolution of 4 cm^−1^, data interval of 2 cm^−1^ and a scan speed of 0.2 cm/s operating in the range of 450–4000 cm^−1^. The ATR sample base plate was equipped with a Diamond ZnSe crystal; an infrared background was collected for all FTIR measurements. Samples (2–5 mg) were placed on the ATR crystal and a pressure was applied to compress the sample in order to obtain the spectra. The IR spectra of CH samples (raw, ball milled, and roller compacted) were examined.

#### 4.2.5. X-ray Powder Diffraction (XRPD), Crystalline Index (ICR) and Degree of Deacetalyion (DDA)

XRPD test was carried out using an X-ray powder diffractometer (Bruker, Karlsruhe, Germany) in 2-theta range of 2–40° 2θ in reflection mode. The X-ray compartment is a D2 Phaser comprising a copper tube, using Kα X-rays of 300 watts of power at 1.54184 Ǻ wavelength. DIFFRAC.SUITE™ computer software was used to analyze the data obtained.

The crystalline index (*I*_CR_) was calculated from the normalized diffractograms. The intensities of the peaks at 110 lattices (*I*_110_, at 2*θ* ≅ 20° corresponding to maximum intensity) and at 2θ ≅ 16° (amorphous diffraction) were used to calculate *I*_CR_ using Equation (3) according to Al Sagheer et al. [22]:

(3) $$I_{\mathrm{CR}}=100\times \left(\;\frac{I_{110}\;-\;I_{am}}{I_{110}}\right)$$

Chitin degree of deacetylation (DDA) is estimated using a method reported Zhang et al. [24], which reported the linear correlation between the crystalline index (*I*_CR_) and the degree of chitin deacetylation (DDA) shown in Equation (4):

(4) $$I_{\mathrm{CR}}\left(\%\right)=103.97-0.7529\;DDA\left(\%\right)$$

#### 4.2.6. Scanning Electron Microscopy (SEM)

The morphology of samples was determined using a Inspect F50 SEM (FEI Company, Eindhoven, The Netherlands), operated at an accelerating voltage from 1–30 kV. Samples (≈0.5 mg) were mounted on graphite tape to an aluminum stub. The powder was then sputter-coated with platinum (Emitech K550X, Qourum Technology, Lewes, UK).

#### 4.2.7. Compression Analysis

CH powder samples (crab, shrimp, squid, and their mixtures) were compressed into tablets using an instrumental single punch bench top tablet press (GTP-1, Gamlen Tablet Press Ltd., Nottingham, UK). Compression was carried out at a punch speed of 60 mm/min by applying five different loads: 100, 200, 300, 400, 500 kg. The samples poured into the die of the GTP had a common weight of 100 ± 1 mg. The diameter of the die was 6 mm. The machine was run by software to display the force-displacement (F-D) curve. Kawakita and Heckel models (Equations (5) and (7), respectively) were utilized to describe the compression analysis of the powders [31,32].

Kawakita analysis describes a linear relationship between the ratio P/C and P, Equation (5), where P is the pressure in MPa, and C is the volume reduction. From the slope and intercept of this relationship, constants ‘*a*’ and ‘*b*’ can be deduced; ‘*a*’ represents the maximum volume reduction that can be attained by the powder. ‘1/*b*’ or ‘*P_K_*’ is another parameter that represents the force required to reduce the powder bed volume to half its maximum value:

(5) $$\frac{P}{C}=\frac{P}{a}+\frac{1}{ab}$$

Volume reduction (*C*) is calculated using Equation (6):

(6) $$C\;=\;1-\frac{\rho_{b}}{\rho_{c}}$$

where $\rho_{b}$ and $\rho_{c}$ are bulk and compact densities (kg/m^3^), respectively.

Heckel analysis describes a relationship between the logarithm of the inverse of compact porosity (*ε*) and the pressure applied, as shown in Equation (7):

(7) $$\ln\frac{1}{\varepsilon }=KP+A$$

Porosity is calculated using the Equation (8):

(8) $$\varepsilon \;=\;1-\rho_{r}$$

where $\rho_{r}$ is the relative density of the compact, and is calculated using the Equation (9):

(9) $$\rho_{r}=\frac{\rho_{c}}{\rho_{T}}$$

where $\rho_{c}$ and $\rho_{T}$ are compact and true densities (kg/m^3^), respectively.

The inverse of the Heckel equation slope or ‘1/*K*’ is an important parameter which assigns Sh5Sq5the type of deformation of materials; whether plastic/elastic or brittle-fracture. This parameter is called the yield pressure and is signified by the symbol ‘*P_Y_*’.

#### 4.2.8. Application of Chitin Excipient Using Metronidazole/Spiramycin as Model Drugs

For dissolution analysis, a sample (100 g) comprising 56.3 g of spiramycin, 30.1 g of metronidazole, 1.9 g of sorbitol powder (solubilizer), and 8.7 g of CH (either CH-Cr, CH-Sh, CH-Sq, or the mixture CH-Sh5Sq5) were granulated with 100 mL of 3% *w*/*w* povidone K30 aqueous solution. The granules were dried at 60 °C for 3 h, then sieved at mesh #18 (particle size = 1 mm). A sample of 415 mg was compressed using a Manesty single punch tablet machine (Manesty F3 single stroke tablet press; West Pharma Services Ltd., Dorset, UK) at an applied force of 35 kN using a 10 mm circular shallow biconvex punch. Rodogyl^®^ tablets were used as a reference.

Apparatus II (USP) dissolution tests were performed using an Erweka DT6 system (Langen, Germany) with paddles running at 50 rpm. 900 mL of 0.1 N HCl was used as the dissolution medium. The amount of drug released was analyzed by measuring the absorbance using a UV spectrophotometer (LABINDIA UV/VIS, UV 3000, Maharashtra, India) at a wavelength of 320 nm for metronidazole, and of 230 nm for spiramycin.

For crushing force and disintegration tests, 415 mg of chitin powder (either CH-Cr, CH-Sh, CH-Sq, or the mixtures CH-Sh9Sq1, CH-Sh7Sq3, and CH-Sh5Sq5) was compressed using the Manesty single punch tablets machine at an applied force of 35 kN using a 10 mm circular shallow biconvex punch. The average crushing force and disintegration time of 10 chitin tablets produced were measured using crushing force tester (Pharma Test PTB 311E. Hainburg, Germany) and disintegration tester (CALEVA, Dorest, UK), respectively.

## 5. Conclusions

Chitin is chemically and pharmacologically inert and can be considered as a future pharmaceutical excipient especially for immediate release tablets, as it can form hard compacts with a very short disintegration time. Depending on the source, CH varies marginally in compression properties. Consequently, CH must be extracted using a single species process. Variations in polymorphic form would not hinder the use of chitin as a pharmaceutical excipient. In order for an excipient comprising chitin to perform ideally in compression and compaction properties, α- and β- polymorphic types would be mixed. In this perspective, α-CH will provide the high bulk density, good flowability, and low water absorption aspects, whereas the β-polymorph will provide the mechanical strength. These aspects were attributed to the difference in crystallinity, fibrous nature, pore size and pore volume of α- and β-CHs.

## Figures and Tables

**Table 1:** True, bulk and tapped densities, HR, and CI of CHs from different sources and their mixtures.

Sample *	Bulk Density (kg/m³)	Tapped Density (kg/m³)	True Density (kg/m³)	HR	CI
**CH-Cr**	332 ± 9	531 ± 9	1400 ± 30	1.60 ± 0.04	60 ± 4
**CH-Sh**	280 ± 6	401 ± 6	1700 ± 40	1.43 ± 0.02	43 ± 2
**CH-Sq**	190 ± 4	338 ± 4	1200 ± 20	1.78 ± 0.03	78 ± 3
**CH-Sh9Sq1**	255 ± 6	402 ± 6	1650 ± 35	1.58 ± 0.03	58 ± 3
**CH-Sh7Sq3**	252 ± 6	403 ± 6	1550 ± 30	1.60 ± 0.03	60 ± 3
**CH-Sh5Sq5**	242 ± 5	403 ± 5	1450 ± 25	1.67 ± 0.02	67 ± 2

* CH-Cr = crab CH, CH-Sh = shrimp CH, CH-Sq = squid CH, CH-Sh9Sq1 = mixture of 90 wt% shrimp CH and 10 wt% squidCH, CH-Sh7Sq3 = mixture of 70 wt% shrimpCH and 30 wt% squid CH, CH-Sh9Sq1 = mixture of 50 wt% shrimp CH and 50 wt% squid CH.

**Table 2:** Water content of CH extracted from different sources.

Sample	CH-Cr	CH-Sh	CH-Sq
**Water content (wt %)**	5.44 ± 0.06	4.84 ± 0.05	8.65 ± 0.06

**Table 3:** Porosity parameters for CH-Cr, CH-Sh, and CH-Sq.

Sample	Pore Volume $\left({\mathrm{cm}}^{3}/g\right)$	Average Pore Diameter (nm)
**CH-Cr**	$(7.308\pm 0.003)\times 10^{-3}$	5.68 ± 0.01
**CH-Sh**	$(6.977\pm 0.003)\times 10^{-3}$	4.32 ± 0.01
**CH-Sq**	$(5.353\pm 0.002)\times 10^{-3}$	3.70 ± 0.01

**Table 4:** Crystalline index (*I*_CR_) and degree of deacetylation (DDA) of CH extracted from different sources.

Sample	CH-Cr	CH-Sh	CH-Sq
***I*_CR_ (%)**	84.3 ± 0.1	85.2 ± 0.1	72.1 ± 0.2
**DDA (%)**	26.1 ± 0.6	24.9 ± 0.5	42.3 ± 0.7

**Table 5:** Kawakita parameters of CH-Cr, CH-Sh, and CH-Sq and their mixtures.

Sample	Slope	Intercept (MPa)	*a* = 1/Slope	*b* = Slope/Intercept (1/MPa)	*ab* (1/MPa)	*P_K_* = 1/*b* (MPa)
**CH-Cr**	1.393 ± 0.066	7.84 ± 0.36	0.7179	0.178	0.127	5.63
**CH-Sh**	1.286 ± 0.061	9.94 ± 0.46	0.7778	0.129	0.101	7.73
**CH-Sq**	1.190 ± 0.056	3.65 ± 0.17	0.8406	0.326	0.274	3.06
**CH-Sh9Sq1**	1.266 ± 0.060	8.46 ± 0.39	0.7899	0.150	0.118	6.68
**CH-Sh7Sq3**	1.251 ± 0.059	8.30 ± 0.38	0.7997	0.151	0.121	6.64
**CH-Sh5Sq5**	1.243 ± 0.059	7.17 ± 0.33	0.8046	0.173	0.139	5.77

**Table 6:** Heckel parameters of CH-Cr, CH-Sh, and CH-Sq and their mixtures.

Sample	Slope (K)	Intercept (A)	$P_{Y}\left(\mathrm{MPa}\right)=1/K$
**CH-Cr**	$6.71\times 10^{-3}$	0.7336	149
**CH-Sh**	$2.69\times 10^{-3}$	0.5739	372
**CH-Sq**	$1.08\times 10^{-2}$	0.8627	93
**CH-Sh9Sq1**	$3.20\times 10^{-3}$	0.5782	306
**CH-Sh7Sq3**	$4.40\times 10^{-3}$	0.6051	227
**CH-Sh5Sq5**	$4.94\times 10^{-3}$	0.6868	202

**Table 7:** Tablet crushing strength.

Sample	CH-Cr	CH-Sh	CH-Sq	CH-Sh9Sq1	CH-Sh7Sq3	CH-Sh5Sq5
**Crushing Strength (*N*)**	54 ± 2	115 ± 3	310 ± 5	121 ± 3	155 ± 4	195 ± 4

**Table 8:** Time for full drug release for metronidazole^®^ (125 mg) and spiramycin^®^ (0.75 M UI) tablets comprising compacted pure CHs from different sources, and CH mixture (CH-Sh5Sq5) compared to Rodogyl^®^ tablets.

Excipient Used in Tablet Preparation	Metronidazole^®^ Complete Dissolution Time (min)	Spiramycin^®^ Complete Dissolution Time (min)
CH-Cr	15	45
CH-Sh	15	30
CH-Sq	30	45
CH-Sh5Sq5	30	45
Rodogyl^®^	30	60

**Table 9:** Composition of CH mixtures (weight %).

Mixture	CH-Sh	CH-Sq
**CH-Sh9Sq1**	90	10
**CH-Sh7Sq3**	70	30
**CH-Sh5Sq5**	50	50
